# Michigan Dental Association

**Published:** 1861-03

**Authors:** J. A. Harris


### Proceedings of Societies.

### MICHIGAN DENTAL ASSOCIATION.

THE sixth annual meeting of the Michigan Dental Association convened in the city of Ann Arbor on Tuesday evening, January 8th, 1861, at 7½ o'clock.

The association was called to order by the President, Dr. Chittenden.

The Secretary being absent, Dr. Harris was, on motion, appointed Secretary *pro tem.*

The Minutes of the last meeting were read and adopted.

The association proceeded to the election of officers for the ensuing year:

Dr. WM. CAHOON, of Detroit, *President.*

Dr. H. BENEDICT, " *Vice-President.*

Dr. J. A. HARRIS, Pontiac, *Rec. and Cor. Secretary.*

Dr. C. B. PORTER, Ann Arbor, *Treasurer.*

Drs. Chittenden, Bancroft, White, *Executive, Examining, and Publishing Committee.*

Owing to the lateness of the hour, the association adjourned to 9 o'clock Wednesday morning.

### MORNING SESSION.

The association was called to order by Dr. Chittenden.

Drs. J. A. Robinson, of Jackson, J. A. Watling, Ypsilanti, were duly elected members.

On motion, Drs. Benedict and Bancroft were appointed to conduct the President elect to the chair, who made a short and eloquent address, thanking the association for the honor conferred upon him. After which, upon receiving an invitation through Dr. Porter, from the Medical Faculty, to visit the State University, adjourned to meet at 2 o'clock.

After spending a short time in museum, called upon Prof. Ford, by whom they were cordially received and entertained, examining many objects of great interest to the profession, from the Professor's own collection of anatomical preparations; witnessed the operation of staphyloraphy by Professor Gunn, which added much to the interest of the visit.

AFTERNOON SESSION.

The regular order of business was taken up—(Reading of Essays.)

Dr. Whiting, on the "Effects of diseased teeth and gums on the general health," being absent, discussion on the subject was opened by

Dr. Porter, who thought that great mischief was done by the neglect of people suffering from this disease, to attend to it, and permitting it to become chronic.

Dr. Robinson did not think he could advance any new ideas, but in such cases, after removing tartar from teeth, scarified gums and used wash of white oak bark; had used it in many cases successfully.

Dr. Porter sometimes used same, with addition of wintergreen.

Dr. Robinson in cases of fungus, removes with knife or scissors that portion, and applies nitrate of silver with brush —related case of an old person whose lower incisors had become very loose; tied the teeth together with silk, and ordered the use of the bark; teeth had become quite firm.

Dr. Geary thought the fetor of diseased teeth and gums very detrimental to health, when taken into the stomach and lungs, as it constantly is, the worst effect being upon the lungs, then through the nervous system acts generally upon the whole body.

Dr. Bancroft thought the bad effects come more through the nervous system than by inhalation. Case of young lady, low state of health, pains in face and mouth, suffering generally from neuralgia; extracted teeth, and cured.

Dr. Chittenden—case; person suffering intensely from neuralgia, gums healthy, teeth good; extracted wisdom tooth and discharged cured; soon returned with same difficulty on other side; extracted wisdom tooth—pain did not return.

Dr. Robinson had not fully made up his mind that diseased teeth and gums always brought on neuralgic affections, persons with offensive breath are more apt to be troubled with catarrhal difficulties; thinks that the malarious effects of the climate is oftener the cause of neuralgia than teeth.

Dr. Harris finds in most cases of offensive breath, the seat of disease is in the nasal organs, but had found some where teeth had become so encased in salivary calculus, as to be no mistake about the cause.

Dr. Geary thinks he can detect the difference between the fetor of the teeth and other diseases of the mouth; wished to correct statement that diseased teeth were never the cause of neuralgia.

Dr. Porter read an essay on "Difficult Dentition." Accepted. Dr. Chittenden—"Essay on Dentition," which was, after some discussion by Drs. Geary, Robinson, and others, accepted.

The subject of Mechanical Dentistry was then taken up, and ably discussed by Dr. Cahoon, who spoke very highly of Dr. F. Y. Clark's method of getting up zinc casts; had used a short time, had no trouble to make a perfect cast.

Dr. Robinson, owing to the shrinkage of zinc, always scraped the surface of impression in the highest part of mouth—this always prevents the plate from rocking.

Dr. Benedict says the shrinkage of zinc is just what we want; a plate that will drop upon and fit the plaster cast, will not stay in the mouth; did not make air chambers, plate would stay better without; introduced a cast of irregular teeth, asking opinions of members as to what would be their method of procedure.

Dr. White would remove molar to make room.

Dr. Geary would fill all that could be saved; have the mouth in as healthy condition as possible; would make rubber plate and regulate; thought could be straightened without loss of any.

Dr. Robinson would make rubber plate, and with wedges would spread and enlarge the whole arch.

Dr. Cahoon—case of irregularity in superior central incisors, shut inside of lower teeth, laterals outside; fitted plate, and with wedges of soft wood moved them outside in two days; thought the case in hand could be remedied with extracting.

Dr. Harris had case similar to this, and commenced the operation without extracting any teeth; the case was straightened, but owing to neglect on part of patient to follow directions and wear plate, the teeth had regained nearly their original position; would in this case extract one tooth on each side, to prevent the tendency to move back.

Dr. Chittenden would take out bicuspid on each side, to make room.

Dr. Robinson had practiced that kind of business for ten years; had come to the conclusion that there was a more excellent way; had in his own family nine cases of irregularity—had treated them all successfully, and thought he knew something about it; would not take out any of the teeth.

Dr. Chittenden thought many of the expensive operations would not pay, as many lost their teeth in this country at an early age.

The Treasurer's report was then read and adopted.

Moved and carried, that Dr. Cahoon's bill be allowed.

Drs. Porter, Robinson, Chittenden, Benedict, Bancroft and White were duly elected delegates to the American Dental Association.

Adjourned to 7 o'clock in evening.

### EVENING SESSION.

Called to order by President, and Dr. Benedict invited to chair, when Dr. Cahoon read an essay on "Dental Etiquette and Fees."

After spirited remarks on the essay by Drs. Chittenden, Robinson, Bancroft, Porter, Geary, and others, was adopted.

On motion,

*Resolved*, That the time of meeting be changed from January to April. *Lost.*

Moved that President appoint committee to select subjects for essays for next meeting. Drs. Robinson, Benedict, and Bartlett were appointed said committee.

Moved and carried, that when we adjourn, it be to meet in the city of Detroit.

The following resolutions were offered by Dr. Chittenden, and unanimously adopted:

*Resolved*, That the thanks of this association be returned to the Faculty of the Michigan Medical University, and particularly to Profs. Ford and Gunn, for their kindness and attendance to the members of this association during its session.

*Resolved*, That a copy of the above be presented to the said Faculty.

The report of the Committee on Essays was then read and adopted:

1. Plugging Teeth.
2. Treatment of diseased Nerves and Fangs.
3. History of Dentistry.
4. Dentistry as a Profession.
5. Springing of Plates.
6. Vulcanite.

On motion, Drs. Chittenden, Bancroft and Geary were appointed committee to appoint essayists. Committee reported the following:

*Plugging Teeth*—Dr. Robinson.

*Treatment of diseased Nerves and Fangs*—Dr. White.

*History of Dentistry*—Dr. Geary.

*Dentistry as a Profession*—Dr. Cahoon.

*Springing of Plates*—Dr. Bartlett.

*Vulcanite*—Dr. Porter.

Letters were read from Dr. Farney, of Detroit, and Dr. Mansfield, of Niles.

Dr. Robinson spoke at length of the importance of rubber to the profession; had used it, and was satisfied that dentures on that material had given better satisfaction than almost any substance.

Dr. Cahoon thought, if there was only one thing to rely upon in the construction of plates, that rubber would be that substance.

Closing remarks were made by Drs. Benedict, Cahoon, Chittenden, Robinson, and others, expressive of their gratification at the good attendance and good feeling that had been manifested, the desire to impart and receive anything pertaining to the interests of the profession,—hoping that when another year has finished its course, and we all convened again under the same auspices, that the results of this session may be seen in the increasing desire to unite more closely the interests of the dental profession of Michigan in one common brotherhood.

On motion,

*Resolved*, That the thanks of this association be tendered to S. D. Palmer, of Cincinnati, for his attendance and assistance in anticipating our wants, with the largest and finest stock of dental goods, instruments and teeth ever brought to Michigan.

On motion, adjourned to meet in the city of Detroit, on Tuesday, the 7th of January, 1862.

J. A. Harris, *Sec'y.*

